# Efficacy and safety of glecaprevir and pibrentasvir in Japanese patients with hepatitis C virus infection aged 75 years or older

**DOI:** 10.1186/s12876-022-02284-z

**Published:** 2022-04-28

**Authors:** Yuri Komaki, Yoshinori Ozono, Kenichi Nakamura, Hisayoshi Iwakiri, Satoru Hasuike, Mitsue Sueta, Tadashi Miike, Shojiro Yamamoto, Hirofumi Uto, Kazunori Kusumoto, Toshimasa Ochiai, Junya Kato, Naoto Komada, Kazuo Kuroki, Toshiharu Eto, Masafumi Shigehira, Shuichi Hirono, Kenji Nagata, Hiroshi Kawakami

**Affiliations:** 1 Division of Gastroenterology and Hepatology, Department of Internal Medicine, Faculty of Medicine, University of Miyazaki, Miyazaki, Japan; 2 Department of Gastroenterology, Miyazaki Medical Center Hospital, Miyazaki, Japan; 3 Department of Internal Medicine, Koga General Hospital, Miyazaki, Japan; 4 Department of Internal Medicine, National Hospital Organization Miyakonojo Medical Center, Miyazaki, Japan; 5 Department of Internal Medicine, Kushima Municipal Hospital, Miyazaki, Japan; 6 Department of Internal Medicine, Shigehira Clinic, Miyazaki, Japan; 7 Department of Internal Medicine, Hirono Naika Clinic, Miyazaki, Japan

**Keywords:** Chronic hepatitis C, Direct-acting antivirals, Glecaprevir, Pibrentasvir, Sustained virological response

## Abstract

**Background:**

It is estimated that approximately 50% of patients with hepatitis C virus (HCV) infection in Japan are currently over 75 years old. However, patients aged ≥ 75 years are typically underrepresented in clinical trials of direct-acting antivirals. This study aimed to evaluate the efficacy and safety of glecaprevir and pibrentasvir (G/P) treatment in Japanese patients with HCV infection aged ≥ 75 years.

**Methods:**

This multicenter, retrospective study included 271 Japanese patients with HCV infection from 12 centers in Miyazaki Prefecture, Japan. Demographic, clinical, virological, and adverse events (AEs) data obtained during and after G/P treatment were collected from medical records. The patients were divided into two groups: younger (n = 199, aged < 75 years) and older (n = 72, aged ≥ 75 years). Virological data and AEs were analyzed according to the age group.

**Results:**

In intention-to-treat (ITT) and per-protocol analyses, the overall sustained virological response 12 (SVR12) rates were 93% and 98.8%, respectively. Two patients in the older group and 14 patients in the younger group dropped out before SVR12 assessment. Although patients in the older group tended to have liver cirrhosis, 95.8% in the older group and 92% in the younger group achieved SVR12 in the ITT analysis (*P* = 0.404). In total, 48 (17.7%) patients experienced treatment-related AEs. Common AEs during treatment included pruritus, headache, and fatigue. The AEs were not significantly different between the two groups.

**Conclusions:**

Compared with younger patients, older patients showed similar virological response and tolerance to G/P treatment.

## Background

Hepatitis C virus (HCV) infection is a significant public health concern and a major cause of liver cirrhosis and hepatocellular carcinoma (HCC) [1, 2]. The prevalence of HCV infection in the general population in Japan is estimated to be 0.9% [3], and the age of patients with HCV infection is gradually increasing [4]. Interferon (IFN)-based treatment was used to treat HCV infection until 2014. Older patients subjected to IFN-based treatment have poor sustained virological response (SVR) rates and high discontinuation rates due to adverse events (AEs) [5, 6].

In 2014, an IFN-free direct-acting antiviral (DAA) treatment regimen was approved for patients with HCV infection in Japan. IFN-free DAA treatments have demonstrated high efficacy, with an improved safety profile and a treatment duration shorter than IFN-based treatment [7, 8]. Several studies have investigated the efficacy and safety of DAA treatment in older patients [9–11].

In 2017, glecaprevir and pibrentasvir (G/P) treatment was approved in Japan to combat HCV infection. G/P is a once-daily, oral, ribavirin (RBV)-free, pangenotypic, IFN-free DAA treatment used to achieve high rates of SVR12 [12, 13]. In addition, G/P treatment has been reported to have a favorable safety profile in patients with genotype 3–6, prior treatment failure, and severe renal impairment, resulting in the need for hemodialysis [14–20]. Although clinical trials on G/P have included patients aged ≥ 65 years, information on patients aged ≥ 75 years is limited. The Japanese population is aging, and it is estimated that approximately 50% of patients with HCV infection are over 75 years old [21]. However, there are few reports regarding the effect of G/P treatment in patients aged ≥ 75 years. To fill this information gap, in this study we assessed the efficacy and safety of G/P treatment in Japanese patients with HCV infection aged ≥ 75 years.

## Methods

### Study design and setting

This was a multicenter, retrospective study conducted at 12 centers in the Miyazaki Prefecture, Japan. A total of 271 consecutive patients with HCV infection who underwent G/P treatment between December 2017 and March 2021 were enrolled in the study. Demographic, clinical, virological, and AE data obtained during and after treatment were retrospectively collected from the medical records. Patients with decompensated liver cirrhosis (Child–Pugh grade B or C), patients with HCV genotypes 4–6, and those with active HCC were excluded from the study. The prevalence of HCV genotype 1 infection is approximately 70%, while that of HCV genotype 2 is approximately 20–30% in Japan [22]. Therefore, HCV genotypes 3–6 account for a small proportion of cases. Absence of active HCC was confirmed by ultrasound, computed tomography scan, or magnetic resonance imaging scan before the initiation of G/P treatment. Overall, 199 patients aged < 75 years were allocated to the younger group and 72 patients aged ≥ 75 years were allocated to the older group. Clinical data were analyzed by group. This study was approved by the Research Ethics Committee of the University of Miyazaki. The procedures used in this study adhere to the tenets of the Declaration of Helsinki.

### Treatment regimens

All patients received 300 mg of glecaprevir and 120 mg of pibrentasvir orally once daily for either 8 or 12 weeks depending on the HCV genotype, the presence or absence of liver cirrhosis, and the history of prior treatment with IFN or other DAA regimens. Hepatologists at each facility clinically diagnosed liver cirrhosis based on laboratory tests and imaging findings, including portosystemic shunt, splenomegaly, or esophageal/gastric varices.

### Follow-up

All patients underwent both physical examination and laboratory testing, which included measurement of HCV RNA levels at baseline and treatment weeks 4, 8 (and 12 for patients with compensated cirrhosis, patients with infected genotype 3, and patients with prior DAA experience) and post-treatment weeks 4, 8, and 12. HCV RNA was measured using the COBAS TaqMan HCV Test (Roche Diagnostics, Tokyo, Japan). The fibrosis-4 (FIB-4) index was calculated before the initiation of G/P treatment. NS5A resistance-associated substitutions (RASs) of HCV were tested by direct sequencing in some patients.

### Definition

SVR 12 was defined as undetectable serum HCV RNA levels 12 weeks after the end of treatment. Virological relapse was defined as undetectable HCV RNA levels by the end of treatment and detectable levels during the follow-up period. Non-virological response was defined as continuously detectable HCV RNA levels during treatment.

### Statistical analysis

Statistical analysis was performed using EZR (Saitama Medical Center, Jichi Medical University, Saitama, Japan), which is a graphical user interface for R (The R Foundation for Statistical Computing, Vienna, Austria). Baseline continuous data were expressed as medians, and categorical data were expressed as numbers and percentages. The effectiveness of G/P treatment was evaluated using intention-to-treat (ITT) and per-protocol (PP) analyses. Univariate analyses were performed using χ^2^, Fisher’s exact, or Mann–Whitney* U* tests. Statistical significance was set at *P* < 0.05.

## Results

### Patient characteristics

**Fig. 1:**
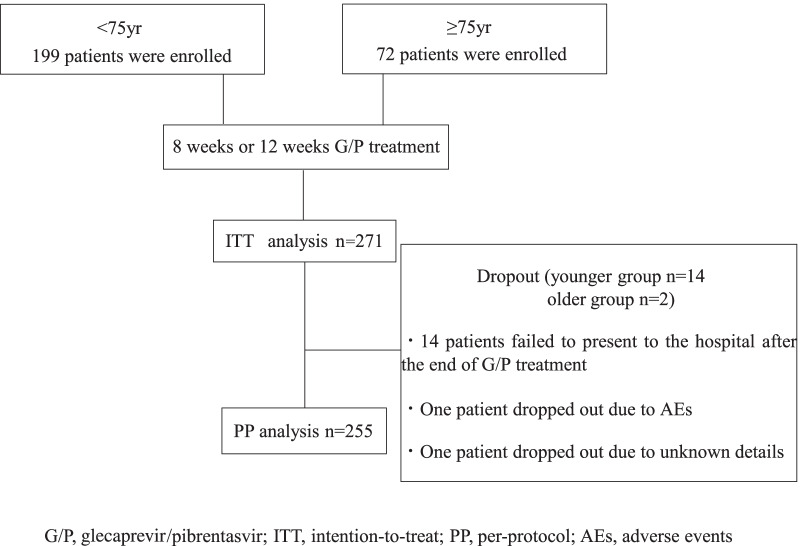
Flowchart of patient selection

This study included 271 patients in total, 16 (5.9%) of whom dropped out during or after the end of the treatment; 14 patients failed to present to the hospital after the end of G/P treatment, one patient dropped out due to AEs, and one patient dropped out due to unknown reason. Thus, ITT and PP analyses were performed for 271 and 255 patients, respectively (Fig. 1).

Table 1 shows the characteristics of the 271 patients enrolled in this study. The median age was 65 years (range, 26–88), and 133 of them (49%) were male. In total, 47 patients (17%) were previously treated with IFN-based and/or IFN-free DAA regimens. Only one patient (aged < 75 years) had HCV genotype 3. Of the 42 patients who were tested for HCV NS5A-RASs before treatment, 29 (69%) were positive. The median HCV viral load prior to treatment was 6.2 log IU/mL (range, 2.3–7.6). The prevalence of cirrhosis and FIB-4 index was higher in the older group. Baseline hemoglobin, platelets, alanine aminotransferase (ALT), and estimated glomerular filtration rate (eGFR) were significantly lower in the older group.

**Table 1:** Baseline characteristics of the participants

Characteristics	Total	< 75 years	≥ 75 years	*P* value
	n = 271	n = 199	n = 72	
Male, n (%)	133 (49)	107 (54)	26 (36)	0.010
Age, median (range), years	65 (26–88)	61 (26–74)	78 (75–88)	< 0.001
Cirrhosis, n (%)	55 (19)	32 (15)	23 (30)	0.004
Hemoglobin, median (range), g/dL	13.8 (8.2–17.2)	13.2 (9.2–17.2)	12.5 (8.2–16.7)	< 0.001
Platelets, median (range), × 10^9^/L,	172 (33–386)	181 (33–386)	148 (35–323)	0.002
AST, median (range), U/L	40 (7–335)	39.5 (7–335)	42 (17–213)	0.516
ALT, median (range), U/L	36 (3–493)	44 (4–493)	32 (3–181)	0.004
eGFR, median (range), mL/min/1.73m^2^	72.6 (3.4–148)	77.2 (3.4–148)	61.4 (7.2–104)	< 0.001
α-fetoprotein, median (range), ng/mL	4.1 (0.9–1782)	4 (0.9–386)	4.3 (1.4–1782)	0.283
FIB-4 index, median (range)	2.6 (0.3–58.4)	2.0 (0.3–18)	3.9 (1.4–58.4)	< 0.001
FIB-4 index ≥ 3.25, n (%)	104 (38)	59 (30)	45 (63)	< 0.001
HCV RNA, median (range), log_10_IU/mL	6.2 (2.3–7.6)	6.3 (2.3–7.6)	6.2 (2.9–7.4)	0.361
HCV genotype (G1/G2/G3/unknown)	143/123/1/4	95/101/1/2	48/22/0/2	0.044
NS5A RASs present, n (%)	29 (69)	18 (62)	11 (85)	0.214
Treatment experienced, n (%)	47 (17)	33 (17)	14 (19)	0.583
Previous HCC treatment, n (%)	15 (6)	9 (5)	6 (8)	0.362

AST, aspartate aminotransferase; ALT, alanine aminotransferase; eGFR, estimated glomerular filtration rate; FIB-4 index, fibrosis-4 index; RASs, resistance-associated substitutions; HCC, hepatocellular carcinoma

### Virological response to G/P treatment

The overall SVR12 rates in the ITT and PP analyses were 93% and 98.8%, respectively. The SVR12 rates in the older group were 95.8% and 98.6%, whereas those in the younger group were 92% and 98.9% in the ITT and PP analyses, respectively. The SVR12 rates in the older group were similar to that in the younger group in both analyses.

**Fig. 2:**
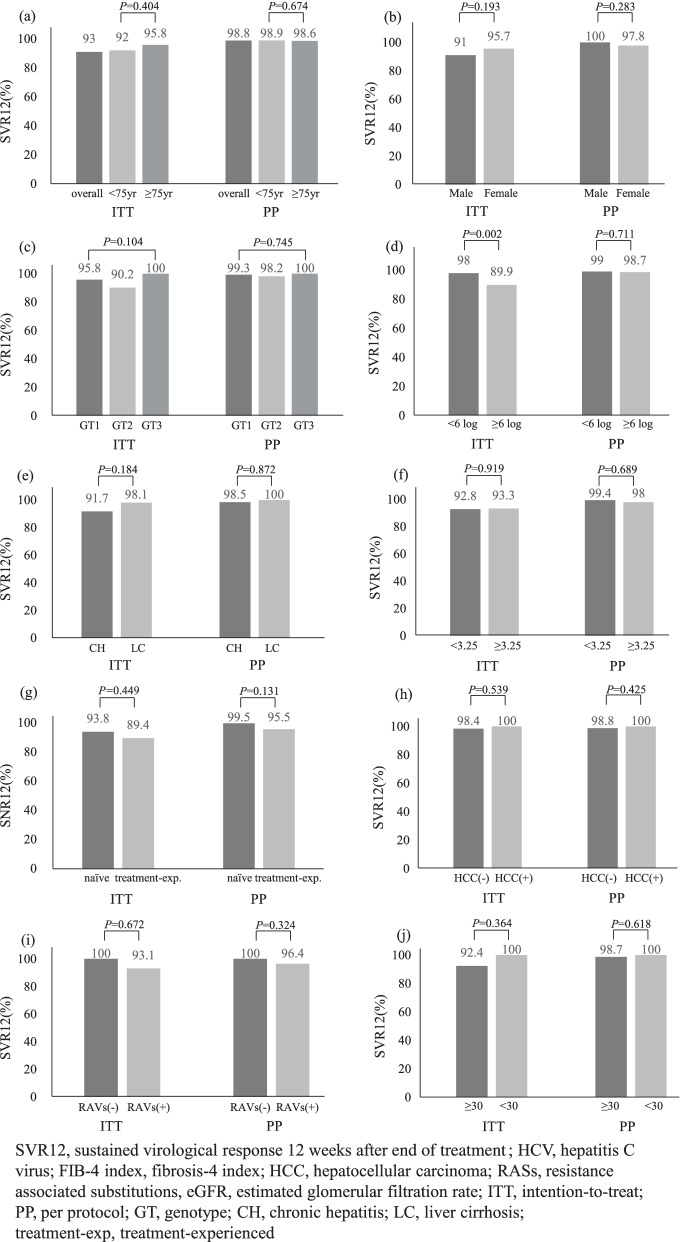
SVR12 rates for each parameter. **a** age, **b** gender, **c** genotype, **d** HCV viral load, **e** liver status, **f** FIB-4 index, **g** prior HCV treatment, **h** history of HCC, **i** presence of NS5A RASs, **j** eGFR

Figure 2 shows the SVR12 rates according to various clinical and demographic factors. In the ITT analysis, the SVR12 rate of patients with an HCV viral load ≥ 6 log IU/mL was significantly lower than that of patients with an HCV viral load < 6 log IU/mL. The SVR12 rates were not significantly influenced by other parameters, such as gender, HCV genotype, liver status, FIB-4 index, prior HCV treatment, history of HCC, presence of NS5A RASs, and eGFR, in either ITT or PP analysis.

Two patients in our study experienced virological relapse (Table 2). One was a 71-year-old woman who underwent Peg-IFN/simeprevir/RBV and daclatasvir/asunaprevir (DCV/ASV) treatment for genotype 1 infection and had NS5A RASs (P32 deletion/Q54H) at baseline. The other patient was a 75-year-old woman who was not tested for NS5A RASs and had no history of prior treatment for genotype 2A infection. One patient in our study experienced a non-virological response. This patient was a 70-year-old woman who had NS5A RASs (S62S/N/T/Y) at baseline and a history of treatment with sofosbuvir/RBV for genotype 2 infection. None of the patients had cirrhosis.

**Table 2:** Characteristics of patients treated with G/P, who did not achieve sustained virological response

No.	Age	Gender	GT	Liver status	History of HCC	Prior HCV treatment	HCV RNA (log_10_IU/mL)	NS5A RAS	Treatment duration (weeks)	Outcome
1	71	F	1	CH	None	Peg-IFN/SMV/RBV DCV/ASV	5.8	P32 deletion/Q54H	12	Relapse
2	75	F	2a	CH	None	None	6.5	Unmeasured	8	Relapse
3	70	F	2	CH	None	SOF/RBV	6.2	S62S/N/T/Y	7	Non-response

F, female; GT, genotype; CH, chronic hepatitis; SMV, simeprevir; RBV, ribavirin; DCV, daclatasvir; ASV, asunaprevir; SOF, sofosbuvir

### Safety and adverse events

The safety profile for G/P treatment is shown in Table 3. In total, 48 (17.7%) patients experienced treatment-related AEs, but none of them were fatal. The most common AEs during treatment were pruritus, headache, and fatigue, which were found in less than 10% of patients, and at similar rates in the older and younger groups. No AEs were significantly different between the older and younger groups. Four patients discontinued G/P treatment prematurely due to AEs, including pruritus, headache, dyspnea, elevation of ALT, cold like symptoms, and elevation of indirect bilirubin. All of them were in the younger group, and eventually achieved SVR12 (Table 4).

**Table 3:** Summary of adverse events

Event, n (%)	Total	< 75 years	≥ 75 years	*P* value
	(n = 271)	(n = 199)	(n = 72)	
Any AEs	48 (17.7)	35 (17.6)	13 (18.1)	0.927
Fatal AEs	0	0	0	
AEs leading to treatment discontinuation	4 (1.5)	4 (2)	0	0.521
Pruritus	22 (8.1)	16 (8)	6 (8.3)	0.862
Headache	6 (2.2)	6 (3)	0	0.306
Fatigue	4 (1.5)	3 (1.5)	1 (1.4)	0.618
Nausea	3 (1.1)	2 (1)	1 (1.4)	0.696
Skin eruption	2 (0.7)	2 (1)	0	0.959
Palpitation	1 (0.4)	1 (0.5)	0	0.595
Abdominal pain	2 (0.7)	2 (1)	0	0.959
Sleepiness	1 (0.4)	1 (0.5)	0	0.595
Hypertension	1 (0.4)	1 (0.5)	0	0.595
Pyelonephritis	1 (0.4)	1 (0.5)	0	0.595
Epigastric discomfort	1 (0.4)	0	1 (1.4)	0.595
Feverish illness	1 (0.4)	0	1 (1.4)	0.595
Head congestion	1 (0.4)	0	1 (1.4)	0.595
Cold-like symptoms	1 (0.4)	1 (0.5)	0	0.595
Dyspnea	1 (0.4)	1 (0.5)	0	0.595
Laboratory abnormalities				
Elevation of total bilirubin	4 (1.5)	1 (0.5)	3 (4.2)	0.101
Elevation of indirect bilirubin	1 (0.4)	1 (0.5)	0	0.595
Elevation of alanine aminotransferase	5 (1.8)	3 (1.5)	2 (2.8)	0.861
Elevation of alkaline phosphatase	2 (0.7)	1 (0.5)	1 (1.3)	0.960
Elevation of creatinine	1 (0.4)	1 (0.5)	0	0.595

Data are expressed as number (%)

AEs, adverse events

**Table 4:** Characteristics and outcome in patients who discontinued G/P treatment

No.	Age	Gender	GT	Liver status	AEs	Treatment duration (weeks)	Outcome	
1	62	M	1b	LC	Pruritus Headache Dyspnea	2	SVR	After the discontinuation of G/P treatment, changing to LDV/SOF
2	63	M	2	CH	Elevation of ALT	6	SVR	
3	72	F	2a	CH	Headache Cold-like symptoms	4	SVR	
4	54	F	2b	LC	Elevation of Total bilirubin	8	SVR	
5	70	F	2	CH	Elevation of ALT	7	Non-response	

M, male; F, female; GT, genotype; LC, liver cirrhosis; CH, chronic hepatitis; AEs, adverse events; ALT, alanine aminotransferase; G/P, glecaprevir/pibrentasuvir; LDV, ledipasvir; SOF, sofosbuvir

## Discussion

In the present study, the overall SVR12 rates in the ITT and PP analyses were 93% and 98.8%, respectively. Those in the older group were 95.8% and 98.6% in the ITT and PP analyses, respectively. There were no significant differences in SVR12 rates between the younger and older groups. Furthermore, there were no significant differences in AEs between the younger and older groups. None of the patients in the older group discontinued G/P treatment due to AEs.

G/P treatment showed a high SVR12 rate in clinical trials [12, 13, 15, 23] and real-world settings [24–26]; however, patients aged ≥ 75 years have typically been under-represented. In Japan, the proportion of undiagnosed HCV carriers is estimated to be highest in the ≥ 75 years age group [27], while it is estimated that approximately 50% of patients with HCV infection are over 75 years old [21]. However, few real-world studies have reported that G/P treatment is highly efficacious and safe in patients aged ≥ 75 years, similar to patients aged < 75 years.

Historically, older patients with HCV have been considered difficult to treat. This is because they have a greater prevalence of advanced liver fibrosis and comorbidities, such as diabetes, hypertension, cardiovascular disease, and severe renal impairment, than younger patients [28, 29]. In the present study, the prevalence of cirrhosis was higher in the older group. Due to the advancement of liver fibrosis, platelet counts, and hemoglobin levels were low and FIB-4 index was significantly higher in the older group. Furthermore, ALT and eGFR were significantly lower in the older group. Akkaya et al*.* reported a significant inverse correlation between HCV viral load and mean ALT levels, and a positive association between ALT levels and duration of HCV infection [30]. In our study, there was no significant difference in the HCV viral load between the older and younger groups. The older group was considered to have a longer duration of HCV infection than the younger group, but their ALT levels were low. We could not identify a definite cause of low ALT levels; however, a previous report showed a similar result [31].

Several of our cases showed highly elevated alpha-fetoprotein (AFP). There were 8 patients with AFP ≥ 100 ng/mL in this study, and one patient with the highest AFP (1782 ng/mL) had a history of HCC. Modest elevations of AFP levels (between 10 and 500 ng/mL and occasionally up to 1000 ng/mL) may also be seen in adult patients with hepatitis of any type or liver cirrhosis. The frequency of elevation (> 10 ng/mL) has been reported as around 20%, in chronic hepatitis and as 40% in cirrhosis. These elevations seem to occur either when there is a high degree of inflammatory activity within the liver, or towards the end of an acute hepatitis when the liver function in recovering [32]. For the aforementioned reasons, AFP was considered to be high, despite excluding cases with active HCC.

HCV infection has been associated with an earlier onset of kidney disease and progression to chronic kidney disease [33], which may be a reason for the low eGFR in the older group. G/P treatment resulted in similarly high SVR12 rates in patients with cirrhosis and severe renal impairment in clinical trials and real-world studies [19, 20, 34]. Likewise, in our study, the SVR12 rates were high, irrespective of liver status and renal impairment.

In our study, the SVR12 rate of patients with an HCV viral load ≥ 6 log IU/mL was significantly lower than that of patients with HCV viral load < 6 log IU/mL only in the ITT analysis. Fifteen out of 169 patients with HCV viral load ≥ 6 log IU/mL and only 1 out of 102 patients with HCV viral load < 6 log IU/mL dropped out of the study. The large number of patients with HCV viral load ≥ 6 log IU/mL resulted in a low SVR12 rate in the ITT analysis.

Two patients experienced virological relapse, and one patient experienced a non-virological response in our study (Table 2). Two of them had NS5A RASs at baseline, and these three patients were infected with genotype 1 or 2. Poordad et al. reported that the SVR12 rates were 79% in cases with past experience with both of NS3/4A protease inhibitor and NS5A inhibitor [35]. A prior Japanese clinical trial showed that G/P treatment is highly effective even for cases with DCV/ASV failure, while the SVR12 rates were 93.9% in patients with prior DAA treatment failure [36]. In addition, Krishman et al*.* reported baseline polymorphisms in NS3 and/or NS5A had no impact on treatment outcomes with G/P treatment [37]. In the same trial, 30 genotype 1b-infected patients had previously received DCV/ASV. Two of these 30 patients had p32 deletions in NS5A at baseline and both of these patients experienced virological failure [36, 37]. Patient No.1 was considered to be relapsed due to p32 deletion. This patient’s RAS was measured at the initiation of G/P treatment, and the attending physician did not know the result when G/P treatment started.

Chen WM et al*.* reported that a high viral load (≥ 10^7^ IU/ml) may predict virological failure in non-cirrhotic patients infected with genotype 2 [38]. The viral load of patient No.2 and No.3 were < 10^7^ IU/ml, and patient No.3 had no p32 deletion. There were no common factors that were obviously associated with virological failure in these patients.

In our study, five patients discontinued G/P treatment; of these, four discontinued due to AEs, and 1 discontinued due to non-virological response to treatment (Table 4). Four cases achieved SVR12, but one of these four patients achieved SVR12 by changing to ledipasvir/sofosbuvir from G/P treatment. Brown et al. and Zamor et al. reported high adherence to G/P treatment as well as high SVR12 rates in those who were not fully adherent to the G/P treatment [39, 40]. The number of cases is small in this study, and as in the previous reports, the SVR12 rates were considered to be high even if the adherence decreases.

Our study has several limitations. First, this was a retrospective study with a small sample size. Second, NS5A RASs could not be tested in all patients, and a few patients failed to achieve SVR12; therefore, we could not correlate NS5A RASs with treatment failure. Third, due to the multicenter, retrospective study, we were unable to obtain sufficient data on some points, including HCV genotype, AFP, and HCV RNA.

## Conclusion

G/P treatment resulted in similarly high virological response and had a good tolerance in older and younger patients and might therefore be effective and safe in patients aged ≥ 75 years.

## Data Availability

The data analyzed for the current study are not publicly available for ethical reasons. All data relevant to the study are included in the article. Anonymized data is available from the corresponding author on request.

## Abbreviations

HCV: Hepatitis C virus
HCC: Hepatocellular carcinoma
IFN: Interferon
SVR: Sustained virological response
AEs: Adverse events
DAA: Direct-acting antiviral
G/P: Glecaprevir/pibrentasvir
RBV: Ribavirin
FIB-4: Fibrosis-4
RASs: Resistance-associated substituitions
ITT: Intention-to-treat
PP: Per-protocol
DCV: Daclatasvir
ASV: Asunaprevir
ALT: Alanine aminotransferase
eGFR: Estimated glomerular filtration rate
AFP: Alpha-fetoprotein
